# SCA-SSST na Emergência: Você Consegue Adivinhar o que está sob o Guarda-Chuva?

**DOI:** 10.36660/abc.20210516

**Published:** 2021-08-09

**Affiliations:** 1 Centro Hospitalar Lisboa Ocidental Hospital de Santa Cruz Departamento de Cardiologia Lisboa Portugal Departamento de Cardiologia, Hospital de Santa Cruz, Centro Hospitalar Lisboa Ocidental, Lisboa - Portugal; 2 Centro Hospitalar Lisboa Ocidental Hospital de Santa Cruz Unidade de Insuficiência Cardíaca Avançada e Transplante Cardíaco Lisboa Portugal Unidade de Insuficiência Cardíaca Avançada e Transplante Cardíaco, Hospital de Santa Cruz, Centro Hospitalar Lisboa Ocidental, Lisboa - Portugal

**Keywords:** Síndrome Coronariana Aguda, Infarto do Miocárdio sem Supradesnível do Segmento ST, Biomarcadores, Isquemia Miocárdica, Troponina, Eletrocardiografia/métodos

O raciocínio por trás da tarefa muitas vezes difícil de decidir se, quando e como tratar o paciente com Doença Cardiovascular (DCV) estabelecida pode ser baseado em duas perguntas simples: (1) Estamos tratando um evento agudo? E (2) Que risco meu paciente corre no futuro? Enquanto a primeira questão implica um tratamento rápido e direto, a segunda premissa depende principalmente da estratificação de risco e, portanto, da capacidade de antecipar a probabilidade de um evento.^1^ Além disso, o médico frequentemente encontra um cenário em que as duas questões devem ser abordadas juntas. Consequentemente, é mais provável que a resposta seja uma probabilidade do que uma resposta categórica (sim ou não). Este é o esquema nos diagnósticos “guarda-chuva”, como nas síndromes coronárias agudas sem supradesnivelamento do segmento ST (SCA-SSST).^2^^,^^3^

Há uma infinidade de ferramentas clínicas na Medicina Cardiovascular^4^^–^^6^ que auxiliam os médicos no processo de tomada de decisão, muitas vezes superando a “suposição aceitável” em cenários agudos.^7^^,^^8^ Entretanto, o uso dessas ferramentas em SCA-SSST é particularmente desafiador por várias razões:

- Em primeiro lugar, isto é realmente um caso de SCA-SSST? O diagnóstico requer a combinação de uma variação do biomarcador cardíaco ao longo do tempo com sintomas isquêmicos miocárdicos ou novos achados de ECG isquêmico, ou imagem de perda de miocárdio viável ou nova anormalidade do movimento regional da parede com um padrão isquêmico.^1^ É importante ressaltar que as troponinas cardíacas de alta sensibilidade facilitaram a identificação de infarto do miocárdio sem supra do segmento ST [particularmente ao reduzir a probabilidade de uma “angina instável” (AI) não identificada com biomarcadores anteriores], mas complicaram um pouco seu diagnóstico diferencial – enfatizando o cenário no qual se deve considerar o termo mais abrangente de lesão miocárdica.^1^^,^^9^ Além disso, os sintomas e alterações do ECG podem ser atribuídos a mecanismos não-isquêmicos, traduzindo-se em sua especificidade medíocre.^9^^,^^10^
- Em segundo lugar, quão grave é a doença subjacente que causa a SCA-SSST? Mesmo quando se considera o Infarto do Miocárdio tipo 1 (espontâneo, relacionado à aterosclerose),^9^ o espectro da doença arterial coronariana (DAC) pode consistir em estenose uniarterial e / ou distal vs. doença proximal grave mais complexa e/ou triarterial. O enredo fica ainda mais complicado se considerarmos os mecanismos fisiopatológicos adicionais que podem estar em ação^11^ – o assim chamado Infarto do Miocárdio do tipo 2.^9^ Nesses casos, notavelmente, a DAC pode estar presente apenas como um agente espectador confundidor.
- Finalmente, como devo tratar este paciente com SCA-SSST? A decisão deve ser baseada nas características clínicas e na gravidade da DAC. Pode envolver abordagem conservadora ou revascularização do miocárdio,^1^ utilizando intervenção coronária percutânea e / ou cirurgia de revascularização do miocárdio (CRM). No entanto, esta última ainda não foi incorporada aos sistemas de escore, mas certamente influencia o prognóstico.^12^^,^^13^

Cedro et al. apresentam um artigo onde os escores clínicos (TIMI, GRACE e HEART) foram utilizados para prever a complexidade da DAC subjacente, de acordo com o escore SYNTAX. Para isso, os autores criaram um estudo observacional de 138 pacientes com SCA-SSST (com média de idade de 60 ± 11 anos, dos quais 68% eram do sexo masculino, e frequentemente apresentando fatores de risco cardiovascular tradicionais). A maioria tinha AI (67,3%) e o espectro de gravidade da DAC era amplo, como se pode inferir pela inclusão de pacientes com doença multiarterial (53,7%) ou ausência de estenose coronariana significativa (> 50%) (29,7%). Os autores descobriram que as correlações entre os escores clínicos e o SYNTAX eram, na melhor das hipóteses, moderadas. Entretanto, o escore HEART teve um desempenho particularmente bom na previsão de DAC complexa (ou seja, escore SYNTAX >32, com uma área sob a curva de 0,81). Curiosamente, um valor de corte > 4 e ≥140 para os escores HEART e GRACE resultou em uma sensibilidade e especificidade de 100% e 97%, respectivamente, para prever essa DAC grave.^14^

Diante dos resultados acima citados, propõe-se que o uso combinado dos escores HEART e GRACE possa ser útil na detecção de DAC complexa.^14^ Deve-se notar, entretanto, que este é um pequeno estudo exploratório unicêntrico, incluindo principalmente pacientes com AI (nos quais o escore GRACE não foi amplamente validado, no que diz respeito ao prognóstico). No entanto, seria uma hipótese atraente investigar se esses escores podem ser incorporados como uma ferramenta válida na via de atendimento de pacientes com SCA-SSST, a saber: (1) Eles poderiam ser utilizados como um novo critério para a lista de estratégia invasiva imediata?, e (2) Isso poderia diferenciar entre os pacientes nos quais a estratégia de pré-tratamento P2Y12 é segura e desejável daqueles nos quais ela pode causar danos (por exemplo, potencialmente retardar a CRM)?

Em conclusão, os resultados preliminares deste estudo sugerem um conceito interessante: em vez de usar as ferramentas clínicas usuais para prever o risco de desfechos, podemos querer utilizá-las para determinar se pode haver uma condição grave e complexa subjacente à DAC, garantindo a revascularização cirúrgica.^15^ Ainda não está claro se essas ferramentas de modelo multivariado de predição de risco podem melhorar os desfechos, mas vale a pena investigar essa hipótese prospectivamente. O trabalho apresentado adiciona um componente pequeno, mas importante, que apoia esse raciocínio, revelando o que pode realmente estar sob o guarda-chuva.
