# Supplementary material for: SCA-SSST na Emergência: Você Consegue Adivinhar o que está sob o Guarda-Chuva?
Source: Arq Bras Cardiol. 2021 Aug 9;117(2):288–9. [Article in Portuguese] doi: 10.36660/abc.20210516 (PMC8395799; doi:10.36660/abc.20210516)
Supplement: Translation

# Translation: NSTE-ACS at the Emergency: Can You Guess What is Under the Umbrella?

**Affiliations:** 1 Lisboa Portugal Departamento de Cardiologia, Hospital de Santa Cruz, Centro Hospitalar Lisboa Ocidental, Lisboa - Portugal; 2 Lisboa Portugal Unidade de Insuficiência Cardíaca Avançada e Transplante Cardíaco, Hospital de Santa Cruz, Centro Hospitalar Lisboa Ocidental, Lisboa - Portugal

**Keywords:** Acute Coronary Syndrome, Non-ST Elevated Myocardial Infarction, Biomarkers, Myocardial Ischemia, Troponin, Electrocardiography/methods

The reasoning behind the often-difficult task of deciding whether, when and how to treat the patient with established Cardiovascular Disease (CVD) might be based on two simple questions: (1) Are we treating an acute event?, and (2) What is my patient at risk of in the future? Whilst the first question implies an expedite straightforward treatment, the second premise depends mostly on risk stratification and, thus, the capability of one to anticipate the likelihood of an event.^1^ Withal, the physician often encounters a scenario in which the two questions must be addressed altogether. Accordingly, the response is more likely one of a probability rather than a categorical (yes or no) answer. This is the outline in umbrella diagnoses, as is non-ST segment elevation (NSTE) Acute Coronary Syndromes (ACS).^2^^,^^3^

There are a multitude of clinical tools in Cardiovascular Medicine^4^^–^^6^ that aid physicians in the decision-making process, often surpassing the “educated guess” in acute settings.^7^^,^^8^ However, the use of these tools in NSTE-ACS is particularly challenging for a number of reasons:

Firstly, is this really NSTE-ACS? The diagnosis requires the combination of a cardiac biomarker variation over time with either myocardial ischemic symptoms or new ischemic ECG findings, or imaging of loss of viable myocardium or new regional wall motion abnormality with an ischemic pattern.^1^ Importantly, high-sensitivity cardiac troponins have facilitated the identification of NSTE Myocardial Infarction [particularly by reducing the likelihood of a missed “unstable angina” (UA) with previous biomarkers] but have somewhat complicated its differential diagnosis – stressing the scenario in which one must consider the more-inclusive myocardial injury term.^1^^,^^9^ Furthermore, symptoms and ECG changes might be attributable to non-ischemic mechanisms, thus translating into their mediocre specificity.^9^^,^^10^

Secondly, how severe is the underlying disease causing NSTE-ACS? Even when considering type-1 (spontaneous, atherosclerosis-related) Myocardial Infarction,^9^ the spectrum of coronary artery disease (CAD) may consist of single-vessel and/or distal stenosis vs. more complex severe proximal and/or three-vessel disease. The plot further thickens if one considers additional pathophysiological mechanisms that can be at work^11^ – the so-called type-2 Myocardial Infarction.^9^ In these cases, notably, CAD might be present merely as a confounding bystander.

Finally, how should I treat this patient with NSTE-ACS? The decision should be based on clinical characteristics and CAD severity. It may involve a conservative approach or myocardial revascularization,^1^ by means of a percutaneous coronary intervention and/or coronary artery bypass grafting (CABG). However, the latter has not been incorporated into score systems but surely influences prognosis.^12^^,^^13^

Cedro et al. present an article where clinical scores (TIMI, GRACE and HEART) were used to predict the complexity of the underlying CAD, as per the SYNTAX score. To do so, the authors designed an observational study enrolling 138 patients with NSTE-ACS (with a mean age of 60 ± 11 years, of whom 68% were males, and often presenting with traditional cardiovascular risk factors). Most had UA (67,3%) and the spectrum of CAD severity was broad, as one may infer from the inclusion of patients with multi-vessel disease (53,7%) or absence of significant (>50%) coronary stenosis (29,7%). The authors have found that the correlations between the clinical and SYNTAX scores were moderate at best. Nonetheless, the HEART score performed particularly well in predicting complex CAD (i.e., SYNTAX >32, with an area under the curve of 0.81). Interestingly, a cut-off value of >4 and ≥140 for HEART and GRACE scores yielded a sensitivity and specificity of 100% and 97%, respectively, to predict such severe CAD.^14^

Given the abovementioned results, it is proposed that the combined use of the HEART and GRACE scores might be useful in detecting complex CAD.^14^ It should be noted, however, that this is a small exploratory single-center study, mostly including patients with UA (in whom the GRACE score has not been extensively validated, as far as prognosis is concerned). Nonetheless, it would be an alluring hypothesis to investigate whether these scores might be incorporated as a valid tool in the pathway of care of NSTE-ACS patients, namely: (1) Could these be used as a novel criterion for immediate invasive strategy listing?, and (2) Could these differentiate between patients in whom P2Y12 pre-treatment strategy is safe and desirable from those in whom it might cause harm (e.g. potentially delaying CABG)?

In conclusion, the preliminary findings of this study suggest an interesting concept: rather than using the usual clinical tools to predict the risk of outcomes, we might want to use them to determine whether there might be a severe complex condition underlying CAD, warranting surgical revascularization.^15^ Whether these multivariate risk prediction model tools might improve outcomes remains unclear, yet this hypothesis is worth being prospectively investigated. The presented work adds a small but important piece supporting this rationale, unveiling what might be truly under the umbrella.
